# Dr. Brown-Séquard

**Published:** 1852-12

**Authors:** 


DR. BROWN-SEQUARD.

During the months of September and October, this gentleman gave highly interesting and successful courses of lectures to two classes of the most distinguished physicians in New York, and is now engaged with equal success in Boston, where his lectures are attended by almost all the eminent physicians of that place.

